# Efficient algorithms to discover alterations with complementary functional association in cancer

**DOI:** 10.1371/journal.pcbi.1006802

**Published:** 2019-05-23

**Authors:** Rebecca Sarto Basso, Dorit S. Hochbaum, Fabio Vandin

**Affiliations:** 1 Department of Industrial Engineering and Operations Research, University of California at Berkeley, Berkeley, CA, USA; 2 Department of Information Engineering, University of Padova, Padova, Italy; 3 Department of Computer Science, Brown University, Providence, RI, USA; 4 Department of Mathematics and Computer Science, University of Southern Denmark, Odense, Denmark; University of Warsaw, POLAND

## Abstract

Recent large cancer studies have measured somatic alterations in an unprecedented number of tumours. These large datasets allow the identification of cancer-related sets of genetic alterations by identifying relevant combinatorial patterns. Among such patterns, *mutual exclusivity* has been employed by several recent methods that have shown its effectiveness in characterizing gene sets associated to cancer. Mutual exclusivity arises because of the complementarity, at the functional level, of alterations in genes which are part of a group (e.g., a *pathway*) performing a given function. The availability of quantitative target profiles, from genetic perturbations or from clinical phenotypes, provides additional information that can be leveraged to improve the identification of cancer related gene sets by discovering groups with complementary functional associations with such targets. In this work we study the problem of finding groups of mutually exclusive alterations associated with a quantitative (functional) target. We propose a combinatorial formulation for the problem, and prove that the associated computational problem is computationally hard. We design two algorithms to solve the problem and implement them in our tool UNCOVER. We provide analytic evidence of the effectiveness of UNCOVER in finding high-quality solutions and show experimentally that UNCOVER finds sets of alterations significantly associated with functional targets in a variety of scenarios. In particular, we show that our algorithms find sets which are better than the ones obtained by the state-of-the-art method, even when sets are evaluated using the statistical score employed by the latter. In addition, our algorithms are much faster than the state-of-the-art, allowing the analysis of large datasets of thousands of target profiles from cancer cell lines. We show that on two such datasets, one from project Achilles and one from the Genomics of Drug Sensitivity in Cancer project, UNCOVER identifies several significant gene sets with complementary functional associations with targets. Software available at: https://github.com/VandinLab/UNCOVER.

## Author summary

Sequencing technologies allow the measurement of somatic alterations in a large number of cancer samples. Several methods have been designed to analyze these alterations, but the characterization of the functional effects of such alterations is still challenging. A recent promising approach for such characterization is to combine alteration data with quantitative profiles obtained, e.g., from genetic perturbations. The analysis of these data is complicated by the extreme heterogeneity of alterations in cancer, with different cancer samples exhibiting vastly different alterations. This heterogeneity is due, in part, to the complementarity of alterations in cancer pathways, with alterations in different genes resulting in the same alteration at the functional level. We develop UNCOVER, an efficient method to identify sets of alterations displaying complementary functional association with a quantitative profile. UNCOVER is much more efficient than the state-of-the-art, allowing the identification of complementary cancer related alterations from genome-scale measurements of somatic mutations and genetic perturbations.

## Introduction

Recent advances in sequencing technologies now allow to collect genome-wide measurements in large cohorts of cancer patients (e.g., [1–6]). In particular, they allow the measurement of the entire complement of somatic (i.e., appearing during the lifetime of an individual) alterations in all samples from large tumour cohorts. The study of such alterations has lead to an unprecedented improvement in our understanding of how tumours arise and progress [7]. One of the main remaining challenges is the interpretation of such alterations, in particular identifying alterations with functional impact or with relevance to therapy [8].

Several computational and statistical methods have been recently designed to identify *driver* alterations, associated to the disease, and to distinguish them from random, *passenger* alterations not related with the disease [9]. The identification of genes associated with cancer is complicated by the extensive *intertumour heterogeneity* [10], with large (100-1000’s) and different collections of alterations being present in tumours from different patients and no two tumours having the same collection of alterations [10, 11]. Two main reasons for such heterogeneity are that i) most mutations are passenger, *random* mutations, and, more importantly, ii) driver alterations target cancer *pathways*, groups of interacting genes that perform given functions in the cell and whose alteration is required to develop the disease. Several methods have been designed to identify cancer genes using *a-priori* defined pathways [12] or interaction information in the form of large interaction networks [13, 14].

Recently several methods (see Section Related work) for the *de novo* discovery of mutated cancer pathways have leveraged the *mutual exclusivity* of alterations in cancer pathways. Mutual exclusivity of alterations, with sets of genes displaying at most one alteration for each patient, has been observed in various cancer types [7, 11, 15, 16]. The mutual exclusivity property is due to the complementarity of genes in the same pathway, with alterations in different members of a pathway resulting in a similar impact at the functional level, while mutations in different members of the same pathway may not provide further selective advantage or may even lead to a disadvantage for the cell (e.g., in synthetic lethality). Even if mutual exclusivity of alterations is neither a sufficient nor a necessary property of cancer pathways, it has been successfully used to identify cancer pathways in large cancer cohorts [15, 17, 18].

**Fig 1:**
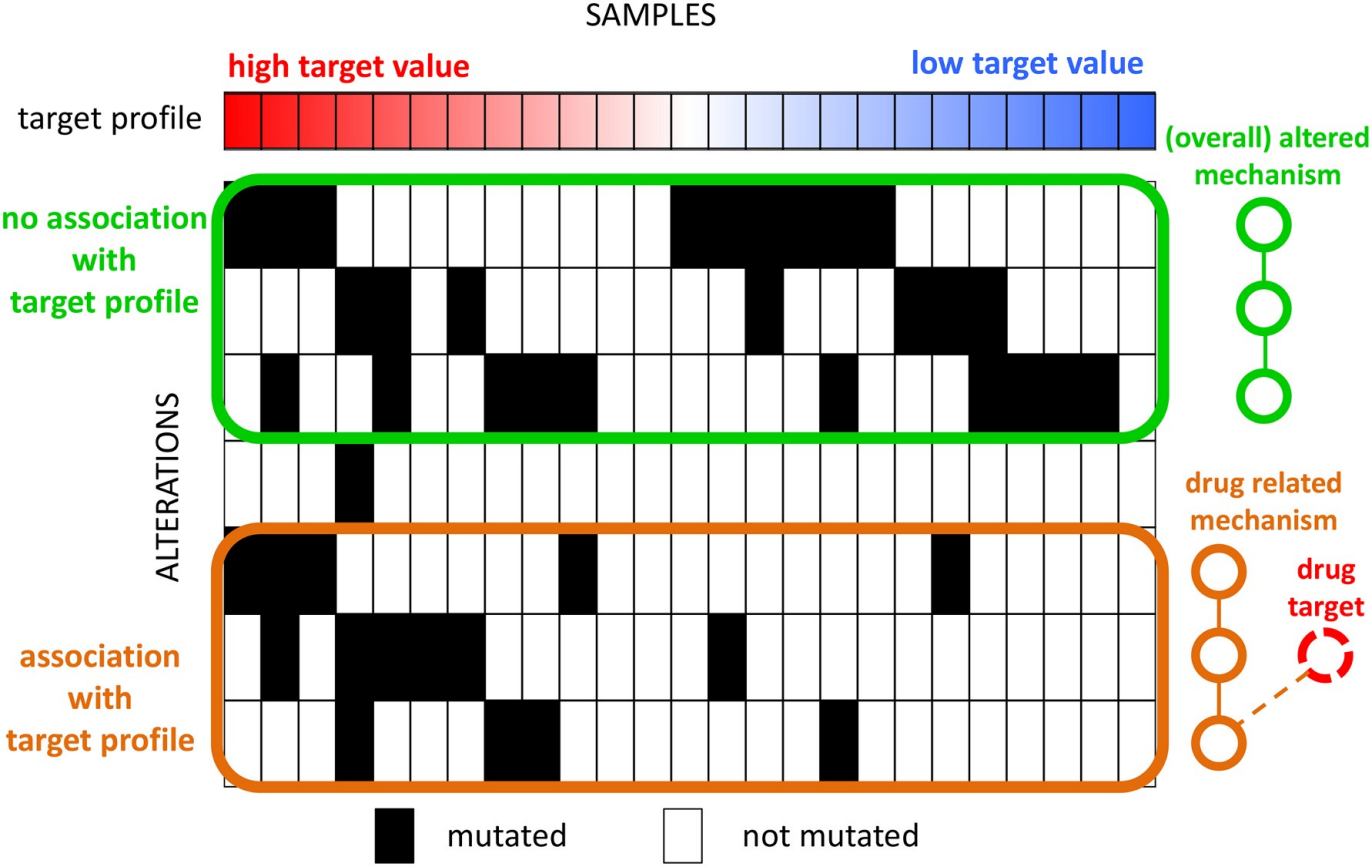
Identification of mutually exclusive alterations associated with a target profile. Alterations in the green set have high mutual exclusivity but no association with the target profile (e.g., a molecular mechanism commonly altered in cancer). Alterations in the orange set have lower mutual exclusivity but strong association with the target profile (e.g., genes in the same pathway of the drug target). Methods that find mutually exclusive sets of alterations without considering the target profile will identify the green set as the most important gene set.

An additional source of information that can be used to identify genes with complementary functions are quantitative measures for each samples such as: functional profiles, obtained for example by genomic or chemical perturbations [19–21]; clinical data describing, obtained for example by (quantitative) indicators of response to therapy; activation measurements for genes or sets of genes, as obtained for example by single sample scores of Gene Set Enrichment Analysis [22, 23]. The employment of such quantitative measurements is crucial to identify meaningful complementary alterations since one can expect mutual exclusivity to reflect in functional properties (of altered samples) that are specific to the altered samples. For example, consider a scenario (Fig 1) in which there are two altered molecular mechanisms: one that is altered in almost all samples and one that is altered in much fewer samples, but is related to the response to a given therapy (for example by interacting with a drug target). Methods that ignore therapy response information will report the first mechanism as significantly altered, while the second mechanisms, altered in a smaller fraction of all samples, is identified only by considering the therapy response information.

### Related work

Several recent methods have used mutual exclusivity signals to identify sets of genes important for cancer [24]. RME [25] identifies mutually exclusive sets using a score derived from information theory. Dendrix [26] defines a combinatorial gene set score and uses a Markov Chain Monte Carlo (MCMC) approach for identifying mutually exclusive gene sets altered in a large fraction of the patients. Multi-Dendrix [27] extends the score of Dendrix to multiple sets and uses an integer linear program (ILP) based algorithm to simultaneously find multiple sets with mutually exclusive alterations. CoMET [18] uses a generalization of Fisher exact test to higher dimensional contingency tables to define a score to characterize mutually exclusive gene sets altered in relatively low fractions of the samples. WExT [18] generalizes the test from CoMET to incorporate individual gene weights (probabilities) for each alteration in each sample. WeSME [28] introduces a test that incorporates the alteration rates of patients and genes and uses a fast permutation approach to assess the statistical significance of the sets. TiMEx [29] assumes a generative model for alterations and defines a test to assess the null hypothesis that mutual exclusivity of a gene set is due to the interplay between waiting times to alterations and the time at which the tumor is sequenced. MEMo [17] and the method from [30] employ mutual exclusivity to find gene sets, but use an interaction network to limit the candidate gene sets. The method by [31] and PathTIMEx [32] introduce an additional dimension to the characterization of inter-tumor heterogeneity, by reconstructing the order in which mutually exclusive gene sets are mutated. None of these methods take quantitative targets into account in the discovery of significant gene sets and sets showing high mutual exclusivity may not be associated with target profiles (Fig 1).

[33] recently developed the repeated evaluation of variables conditional entropy and redundancy (REVEALER) method, to identify mutually exclusive sets of alterations associated with functional phenotypes. REVEALER uses as objective function (to score a set of alterations) a re-scaled mutual information metric called *information coefficient* (IC). REVEALER employs a greedy strategy, computing at each iteration the conditional mutual information (CIC) of the target profile and each feature, conditioned on the current solution. REVEALER can be used to find sets of mutually exclusive alterations starting either from a user-defined seed for the solution or from scratch, and [33] shows that REVEALER finds sets of meaningful cancer-related alterations.

### Our contribution

In this paper we study the problem of finding sets of alterations with complementary functional associations using alteration data and a quantitative (functional) target measure from a collection of cancer samples. Our contributions in this regard are fivefold. First, we provide a rigorous combinatorial formulation for the problem of finding groups of mutually exclusive alterations associated with a quantitative target and prove that the associated computational problem is NP-hard. Second, we develop two efficient algorithms, a greedy algorithm and an ILP-based algorithm to identify the set of *k* genes with the highest association with a target; our algorithms are implemented in our method fUNctional Complementarity of alteratiOns discoVERy (UNCOVER). Third, we show that our algorithms identify highly significant sets of genes in various scenarios; in particular, we compare UNCOVER with REVEALER on the same datasets used in [33], showing that UNCOVER identifies solutions of higher quality than REVEALER while being on average two order of magnitudes faster than REVEALER. Interestingly, the solutions obtained by UNCOVER are better than the ones obtained by REVEALER even when evaluated using the objective function (IC score) optimized by REVEALER. Fourth, we show that the efficiency of UNCOVER enables the analysis of large datasets, and we analyze a large dataset from Project Achilles, with thousands of genetic dependencies measurements and tens of thousands of alterations, and a large dataset from the Genomics of Drug Sensitivity in Cancer (GDSC) project, with hundreds of drug sensitivity measurements and tens of thousands of alterations. On such datasets UNCOVER identifies several statistically significant associations between target values and mutually exclusive alterations in genes sets, with an enrichment in well-known cancer genes and in known cancer pathways.

## Materials and methods

This section describes the problem we study and the algorithms we designed to solve it, that are implemented in our tool UNCOVER. We also describe the data and computational environment for our experimental evaluation.

### UNCOVER: Functional complementarity of alterations discovery

**Fig 2:**
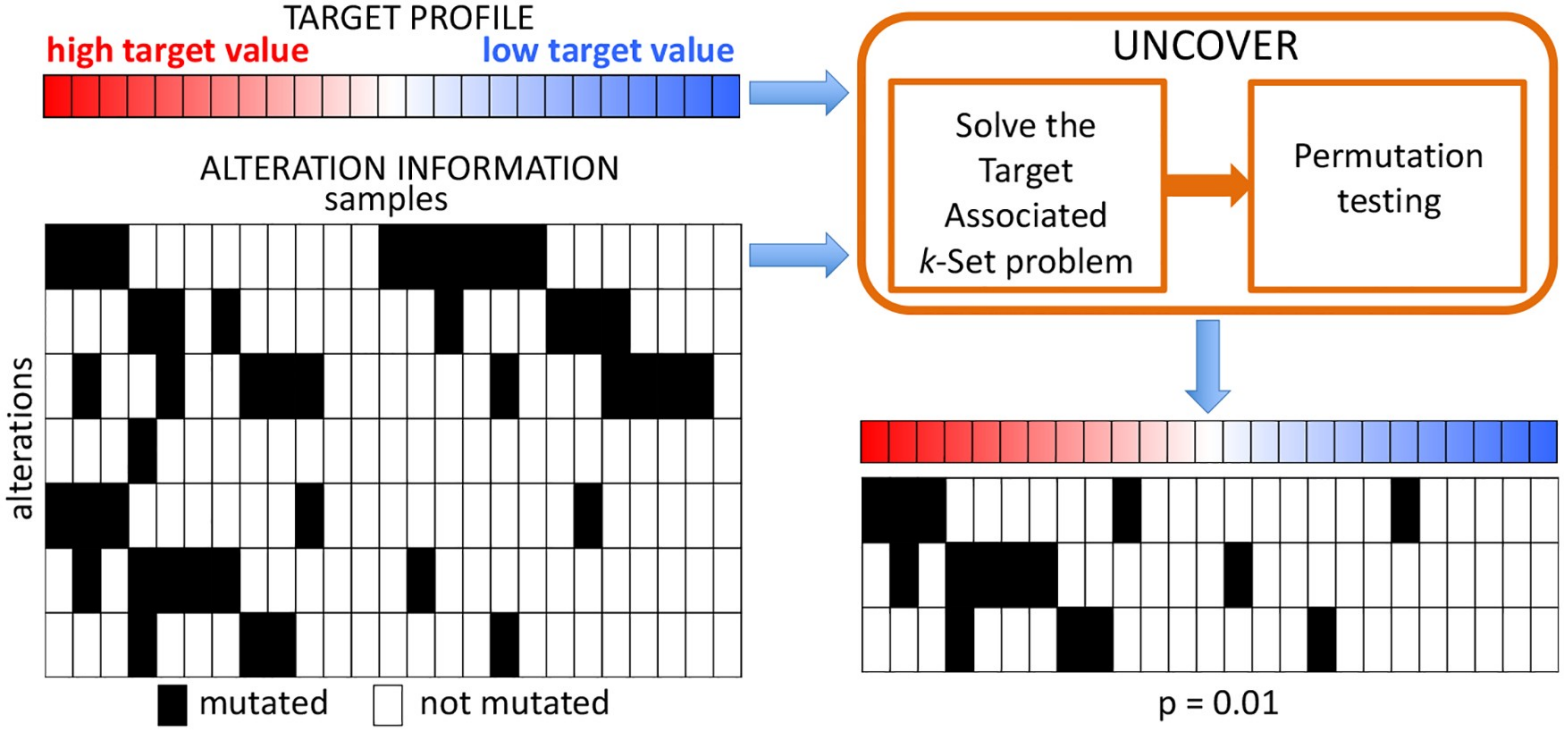
UNCOVER: Functional complementarity of alterations discovery. UNCOVER takes in input the alterations information and a target profile for a set of samples, and identifies the set of complementary alterations with the highest association to the target by solving the Target Associated *k*-Set problem and performing a permutation test.

The workflow of our algorithm UNCOVER is presented in Fig 2. UNCOVER takes in input information regarding 1. the alterations measured in a number of samples (e.g., patients or cell lines), and 2. the value of the *target* measure for each patient. UNCOVER then identifies the set of mutually exclusive alterations with the highest association to the target, and employs a permutation test to assess the significance of the association. Details regarding the computational problem and the algorithms used by UNCOVER are described in the following sections. The implementation of UNCOVER is available at https://github.com/VandinLab/UNCOVER.

#### Computational problem

Let *J* = {*j*_1_, …, *j*_*m*_} be the set of samples and let *G* = {*g*_1_, …, *g*_*n*_} be the set of genes for which we have measured alterations in *J*. We are also given a *target profile*, that is for each sample *j* ∈ *J* we have a target value $w_{j}\in R$ which quantitatively measures a functional phenotype (e.g., pathway activation, drug response, etc.). For each sample *j* ∈ *J* we also have information on whether each *g* ∈ *G* is altered or not in *j*. Let *A*_*g*_ be the set of patients in which gene *g* ∈ *G* is mutated. We say that a patient *j* ∈ *J* is covered by gene *g* ∈ *G* if *j* ∈ *A*_*g*_ i.e. if gene *g* is mutated in sample *j*. Given a set of genes *S* ⊂ *G*, we say that sample *j* ∈ *J* is covered by *S* if *j* ∈ ∪_*g* ∈ *S*_ *A*_*g*_.

The goal is to identify a set *S* of at most *k* genes, corresponding to *k* subsets *S*_1_, *S*_2_, …*S*_*k*_ where for each subset *S*_*i*_ we have that *S*_*i*_ ⊆ *J*, such that the sum of the weights of the elements covered by *S* is maximized. We also penalize overlaps between sets when an element is covered more than once by *S* by assigning a penalty *p*_*j*_ for each of the additional times *j* is covered by *S*. As penalty we use the positive average of the normalized target values if the original weight of the element was positive. If the original weight of the element was negative we assign a penalty equal to its weight.

Let *c*_*S*_(*j*) be the number of sets in *S*_1_, …, *S*_*k*_ that cover element *j* ∈ *J*. Therefore for a set *S* of genes, we define its weight *W*(*S*) as:

$$\begin{array}{c} W\left(S\right)=\sum_{j\in \cup_{s\in S}A_{s}}w_{j}-\sum_{j\in \cup_{s\in S}A_{s}}\left(c_{S}\left(j\right)-1\right)p_{j} \end{array}$$

#### The target associated *k*-set problem

*G*iven a set *J* of samples, sets $A_{g_{1}},\ldots ,A_{g_{n}}$ describing alterations of genes *G* = {*g*_1_, …, *g*_*n*_} in the set *J*, weights *w*_*j*_ and penalties *p*_*j*_ > 0 for each sample *j* ∈ *J* find the *S* of ≤ *k* elements maximizing *W*(*S*).

The following result defines the computational hardness of the problem above.

**Theorem 1**. *The Target Associated k-Set problem is NP-hard*.

*Proof*. The proof is by reduction from the Maximum Weight Submatrix Problem (MWSP) defined and proved to be NP-hard in [34]. The MSWP takes as input an *m* × *n* binary matrix *A* and an integer *k* > 0 and requires to find the *m* × *k* column sub-matrix $\hat{M}$ of *A* that maximizes the objective function |Γ(*M*)| − *ω*(*M*), where Γ(*M*) is the set of rows with at least one 1 in columns of *M* and *ω*(*M*) = ∑_*g* ∈ *M*_|Γ({*g*})| − |Γ(*M*)|.

Given an instance of Maximum Weight Submatrix Problem, we define an instance of the Target Associated *k*-Set as follow: the set of samples *J* corresponds to the rows of *A*, the set of genes *G* corresponds to the columns of *A*, and the set *S*_*g*_ of samples covered by gene *g* ∈ *G* is the subset of the rows in which *g* has a 1. By setting *w*_*j*_ = 1 and *p*_*j*_ = 1 for all *j* ∈ *J*, we have that the objective function of MWSP corresponds to the weight *W*(*S*) for the Target Associated *k*-Set therefore the optimal solution of the Target Associated *k*-Set corresponds to the optimal solution of MWSP.

#### ILP formulation

In this subsection we provide an ILP formulation for the Target Associated *k*-Set problem. Let *x*_*i*_ be a binary variable equal to 1 if set *i* ∈ *G* is selected and *x*_*i*_ = 0 otherwise. Let *z*_*j*_ be a binary variable equal to 1 if element *j* is covered and *z*_*j*_ = 0 otherwise. Let *y*_*j*_ denote the number of sets in the solution covering element *j*. Finally, let *w*_*j*_ be the weight of element *j* and *p*_*j*_ be the penalty for element *j*

In our ILP formulation, the following constraints need to be satisfied by a valid solution:

- the total number of sets in the solution is at most *k*: ∑_*i*_ *x*_*i*_ ≤ *k*
- for each element *j* ∈ *J* we have: $y_{j}=\sum_{i:j\in S_{i}}x_{i}$
- for each element *j* ∈ *J*, if *j* is covered by the current solution then the number of times *j* is covered in the solution is at least 1: *y*_*j*_ ≥ *z*_*j*_
- for each element *j* ∈ *J*, if *j* is covered by at least one element in the current solution then *j* is covered: *z*_*j*_ ≥ *y*_*j*_/*k*.

With the variables defined above, the score for a given solution is

$$\begin{array}{c} z\left(q\right)=\max\sum_{j=1}^{m}\left(w_{j}+p_{j}\right)z_{j}-\sum_{j=1}^{m}p_{j}y_{j}. \end{array}$$ (1)

*z*(*q*) constitutes the objective function of our ILP formulation.

#### Greedy algorithm

Since solving ILPs can be impractical for very large datasets, we also design a *k*-stage greedy algorithm to solve the Target Associated *k*-Set problem. During each stage the algorithm picks 1 set *A*_*i*_ to be part of the solution; this is done by first computing the total weight of each subset which is defined as the sum of the weights of its elements $W\left(A_{i}\right)=\sum_{j\in A_{i}}w_{j}$. Then the algorithm finds the subset *A*_*i*_ of maximum positive weight and adds it to the solution. It may be that at some stage *ℓ* no additional set of positive weight can be selected, in this case, the solution obtained after stage *ℓ* − 1 will be our output. At the end of the iteration the weight of every element *j* that belonged to the chosen set *A*_*i*_ is set to the negative of penalty *p*_*j*_, in order to penalize future selections of the same elements. The greedy algorithm is described in Algorithm 1.

**Algorithm 1**: Greedy Coverage

**Input**: A set of elements *J* (samples), a class *I* of subsets of *J* (genetic alterations) and an integer *k* (maximum number of alterations in the solution). Each element *j* ∈ *J* has an associated weight *w*_*j*_ (target profile) and a penalty *p*_*j*_.

**Output**: *k* subsets *S*_1_, *S*_2_, …*S*_*k*_, where each subset selected is a member of *I*, such that the sum of the weights of the elements in the selected sets is maximized and the overlap between selected sets is minimized.

**for** *ℓ* ← 1 *to k* **do**

 **for** *i* ← 1 *to n* **do** $W\left(A_{i}\right)\leftarrow \sum_{j\in A_{i}}w_{j}$;

 $S_{l}\leftarrow \text{arg}\;{\text{max}}_{W\left(A_{i}\right)>0}\left\{W\left(A_{i}\right)\right\}$;

 **for** *j* ∈ *S_ℓ_* **do** *w*_*j*_ ← −*p*_*j*_;

**end**

**return** *S*_1_…*S*_*k*_;

We note that our greedy algorithm is analogous to the greedy algorithm for the Maximum k-Coverage problem [35] with the difference that rather than eliminating the elements already selected we change their weight to a penalty. Also, assuming it is acceptable to return less than *k* sets, we only pick a set if it has a positive weight. The running time of the algorithm is *O*(*kmn*) where *m* = number of samples and *n* = number of alterations.

While the greedy algorithm may not return the optimal solution, we prove that it provides guarantees on the weight of the solution it provides.

**Proposition 1**. Let S* *the optimal solution of the Target Associated k-Set and* $\hat{S}$ *be the solution returned by the greedy algorithm. Then* $W\left(\hat{S}\right)\geq W\left(S^{*}\right)/k$.

*Proof*. Note that the weight of subsets in the optimal solution *W*(S*) can only be lower compared to the original weight of the subsets, since the only weight update operation performed is to substitute positive weights of elements selected with a negative penalty.

The first subset ${\hat{S}}_{1}$ selected by our algorithm is the set of maximum weight out of all subsets and therefore $W\left({\hat{S}}_{1}\right)\geq W\left(S_{\ell }^{*}\right)$ for *ℓ* = 1…*k*. By the pigeonhole principle, one of these subsets in the optimal solution must cover at least *W*(S*)/*k* worth of elements. Thus $W\left({\hat{S}}_{1}\right)\geq W\left(S^{*}\right)/k$. Therefore the first subset selected by the algorithm already gives a 1/*k* approximation of the optimal solution. In subsequent iterations of the algorithm we only pick additional sets if they have a positive weight so our approximation ratio can only improve.

We also prove that the bound above is tight.

**Proposition 2**. *There are instances of the Target Associated k-Set such that* $W\left(\hat{S}\right)=W\left(S^{*}\right)/k$.

The proof is in S1 Appendix.

While the proposition above is based on an extreme example, our experimental analysis shows that in practice the greedy algorithm works well and often identifies the optimal solution. We therefore analyze the greedy algorithm under a generative model in which there is a set *H* of *k* genes with mutually exclusive alterations associated with the target, while each gene *g* ∈ *G* \ *H* is mutated in sample *j* with probability *p*_*g*_ independently of all other events. We also assume that the weights *w*_*j*_ are such that ∑_*j* ∈ *J*_ *w*_*j*_ = 0 and for each *j*: |*w*_*j*_ ≤ 1|. (In practice this is achieved by normalizing the target values before running the algorithm, by subtracting to each *w*_*j*_ the average value ∑_*j* ∈ *J*_ *w*_*j*_/*m* and then dividing the result by the maximum of the absolute values of the transformed *w*_*j*_’s). Note that this last condition implies that |*p*_*j*_|≤1 for all *j*. We also assume that for genes in *H* the following assumptions hold:

- the set *H* has an association with the target, i.e.: $E\left[W\left(H\right)\right]\geq \frac{m}{c^{\prime }}$ for a constant *c*′ ≥ 1.
- each gene of *H* contributes to the weight of *H*, i.e. for each *S* ⊂ *H* and each *g* ∈ *H* \ *S* we have $E\left[W\left(S\cup \left\{g\right\}\right)\right]-E\left[W\left(S\right)\right]\geq \frac{W(H)}{kc^{{}^{\prime \prime }}}$ for a constant *c*″ ≥ 1.

The following shows that, if enough samples from the generative model are considered, the greedy algorithm finds the set *H* associated with the target with high probability.

**Proposition 3**. *If m* ∈ Ω(*k*^2^ ln(*n*/*δ*)) *samples from the generative model above are provided to the greedy algorithm, then the solution of the greedy algorithm is H with probability* ≥ *δ*.

The proof is in S1 Appendix.

#### Statistical significance

To assess the significance of the solution reported by our algorithms we use a permutation test in which the dependencies among alterations in various genes are maintained, while the association of alterations and the target is removed. In particular, a permuted dataset under the null distribution is obtained as follows: the sets $A_{g},g\in G$ are the same as observed in the data; the values of the target are randomly permuted across the samples.

To estimate the *p*-value for the solutions obtained by our methods we used the following standard procedure: 1) we run an algorithm (ILP or greedy) on the real data D, obtaining a solution with objective function $o_{D}$; 2) we generate *N* permuted datasets as described above; 3) we run the same algorithm on each permuted dataset; 4) the *p*-value is then given by (*e* + 1)/(*N* + 1), where *e* is the number of permuted datasets in which our algorithm found a solution with objective function $\geq o_{D}$.

### Data and computational environment

#### Alteration data

We downloaded data for the Cancer Cell Line Encyclopedia on 25^*th*^ September, 2017 from http://www.broadinstitute.org/ccle. In particular we used the mutation (single nucleotide variants) and copy number aberrations (CNAs) which are derived from the original Cancer Cell Line Encyclopedia (CCLE) mutations and CNA datasets. The file we used is CCLE_MUT_CNA_AMP_DEL_0.70_2fold.MC.gct. It consists of a binary (0/1) matrix across 1,030 samples and 48,270 gene alterations in the form of mutations, amplifications and deletions, with a 1 meaning that the alteration is present in a sample, and a 0 otherwise. For the GDSC experiments [36, 37], we used the alteration provided at https://depmap.org/portal/download/all/. We downloaded the data on July 6^*th*^ 2018. In particular we used mutation data from portal-mutation-2018-06-21.csv that includes binary entries for 18652 mutations. Additionally we considered 22746 amplifications and 22746 deletions computed from the gene copy number data in portal-copy_number_relative-2018-06-21.csv, with an amplification defined by a copy number above 2 and a deletion defined by a copy number below -1.

#### Target data

In terms of target values we use the same datasets used by [33] to compare the performance of UNCOVER with REVEALER. In particular we used the following files available through the Supplementary Material of [33]: CTNBB1_transcriptional _reporter.gct, which consists of measurements of a *β*-catenin reporter in 81 cell lines; NFE2L2_activation_profile.gct, which includes NFE2L2 enrichment profiles for 182 lung cell lines; MEK_inhibitor_profile.gct, which contains MEK-inhibitor PD-0325901 sensitivity profile in 493 cancer cell lines from the Broad Novartis CCLE14l; and KRAS_essentiality_profile.gct, which corresponds to the feature KRAS from a subset of 100 cell lines from the Achilles project dataset. In all these cases we considered the same direction of association (positive or negative) between alterations and the target as in [33]. Since our algorithm is very efficient we then decided to run it on a large dataset on genetic dependencies from Project Achilles (https://portals.broadinstitute.org/achilles), that uses genome-scale RNAi and CRISPR-Cas9 perturbations to silence or knockout individual genes. In particular, we use the whole 2.4.2 Achilles dataset (Achilles_QC_v2.4.3.rnai.Gs.gct) available from the project website. This dataset provides phenotype values for 5711 targets, corresponding to genes silenced by shRNA. The phenotype values correspond to ATARiS [38] gene (target) level scores, quantifying the cell viability when the target gene is silenced by shRNA. These scores are provided for 216 cell lines [19], with 205 of them appearing in CCLE. We also used UNCOVER to analyze a large datasets from the Genomics of Drug Sensitivity in Cancer (GDSC) project (https://www.cancerrxgene.org/) which provides drug sensitivity data generated from high-throughput screening using fluorescence-based cell viability assays following 72 hours of drug treatment. In particular, we considered the area under the curve for each experiment as target. These scores are provided in the file portal-GDSC_AUC-2018-06-21.txt, available trough the DepMap portal (https://depmap.org) [39] for 265 compounds and 743 cell lines, with 736 having alteration data in DepMap.

#### Data preprocessing

To be consistent with REVEALER we discarded features with high or low frequency, in particular features present in less than 3 samples or more than 50 samples were excluded from our analyses. The only exception was the MEK-inhibitor example, where the high frequency threshold was changed to be 100 since the number of original samples was substantially higher (i.e., 493) for this case. From the Achilles dataset we excluded targets that have at least one missing value, in particular in this case we exclude 21 of the 5711 sets of target scores. From the GDSC dataset, since many samples have at least one target with a missing value, for every target we excluded samples with missing value for that target, that results in a different number of samples for each target. The number of samples varied between 240 and 705. We filtered alterations to only have alterations with frequencies between 0.1 and 0.25, removing in this way genes that have high alteration frequency due to genomic features not important for to the disease (e.g., gene length) [9]. In all our experiments we normalized the target values before running the algorithm, by subtracting to each weight *w*_*j*_ the average value ∑_*j* ∈ *J*_ *w*_*j*_/*m* and dividing the result by the standard deviation of the (original) *w*_*j*_’s, in order to have both positive and negative target values.

#### Simulated data

We investigated how effective UNCOVER is at finding selected alterations in a controlled setting, where the ground truth is known. We generated target values according to a normal distribution with mean 0 and standard deviation 1. We tested dataset with 200, 600, 1000 and 10000 samples. For each dataset we considered the 38002 gene alterations present in CCLE and for each of them we placed alterations in the samples independently of all other events with the same frequencies as they appear in CCLE. To be consistent with the preprocessing done on real data we filtered alterations to only have alterations with frequencies between 0.1 and 0.25. We also generated a set *T* of 5 features to have an association with the target values. This association was varied throughout the experiments to cover different percentages of positive and negative targets. In particular we generated the selected features to cover 100%, 80%, 60%, 40% of the positive target values and 5%, 10%, 15%, 20% of the negative target values respectively, choosing random subsets of samples with positive or negative target values. We will refer to the parameter indicating the percentage of samples with positive target values selected as *P* and to the parameter for the percentage of samples with negative target values selected as *N*. We divided the number of targets covered by each of the 5 mutations equally.

#### Computing environment and solver configuration

To describe and solve an ILP we used AMPL 20150516 and CPLEX 12.6.3. All parameters in CPLEX were left at their default values. We implemented our greedy algorithm in Python 3.6.1. We run our experiments on the same datasets considered by REVEALER [33] and on the Achilles project dataset on a MacBook Air with 1.7 GHz Intel Core i7 processor, 8 GB RAM and 500 GB of local storage. Experiments on simulated data were conducted on local nodes of a computing cluster. Each node had the following configuration: four 2.27 GHz CPUs, 5.71 GB RAM and 241 GB of storage. Experiments on the GDSC dataset for UNCOVER and REVEALER were conducted on an iMac with 3.4 GHz Intel Core i5 processor and 16 GB RAM. For the time comparison between UNCOVER and REVEALER we run the R code provided in [33] on the same machine used for UNCOVER, using R 3.5.1. All the parameters were left at their given values except for the number of permutations used to calculate their p-value, which we changed in order to compare the running time of the methods excluding the time needed to compute *p*-values.

## Results and discussion

We tested UNCOVER on a number of cancer datasets in order to compare its results to the ones obtained without using the target, to state-of-the-art algorithms, and to test whether UNCOVER allows the analysis of large datasets. In particular, we first assessed the impact of the target values on the results of UNCOVER. We then compared UNCOVER with REVEALER using four datasets described in [33] as well as the GDSC project dataset described above. We then used simulated data to asses the performance of UNCOVERin finding groups of alterations associated with a target. We then performed a scalability test using a large dataset from the Achilles project and alterations from the Cancer Cell Line Encyclopedia (CCLE). Finally, we used UNCOVER to analyze a drug sensitivity dataset from the GDSC project.

### Impact of target

**Fig 3:**
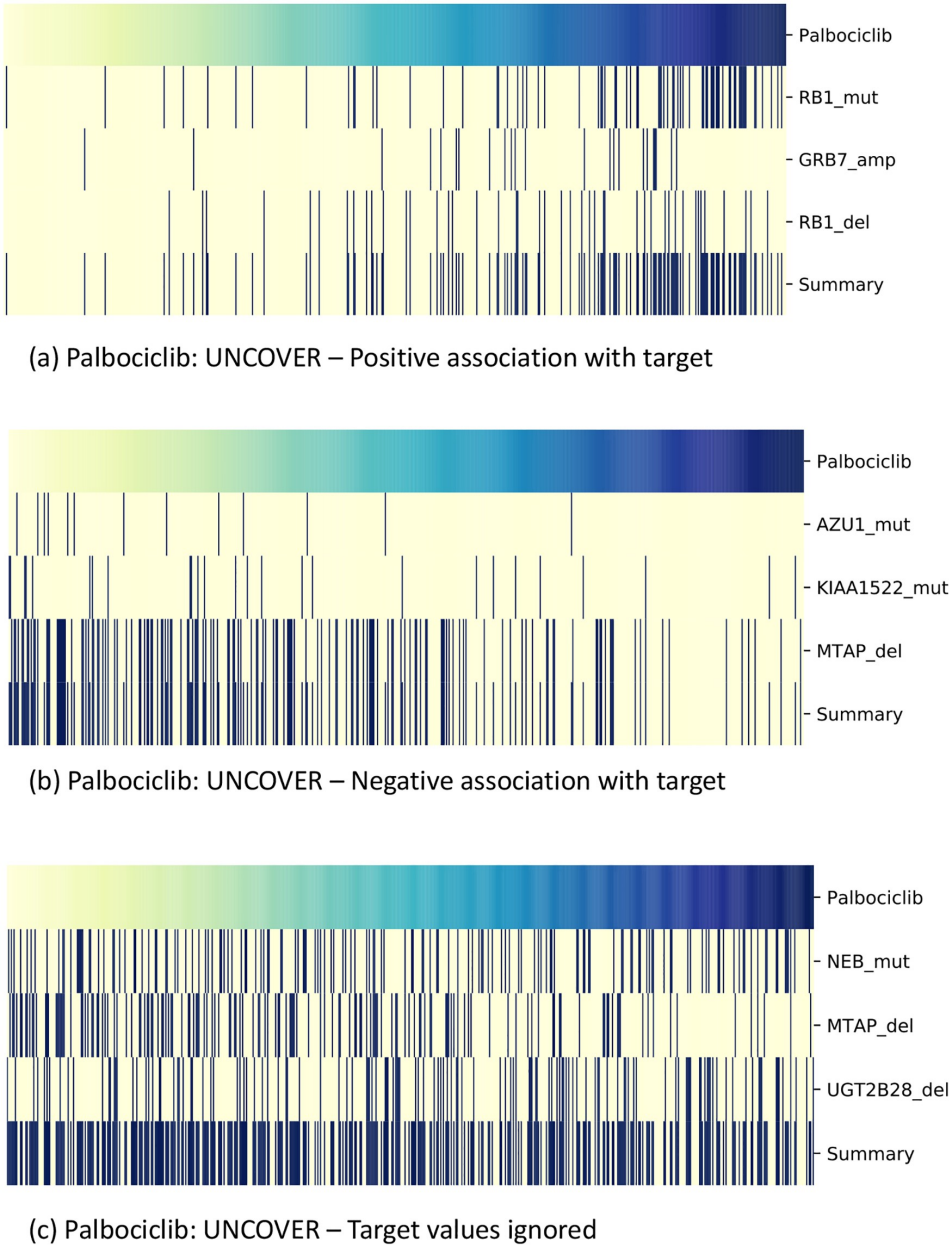
Impact of the target on the results. UNCOVER results for target Palbociclib considering or ignoring target values. (a) Solution found by UNCOVER looking for an association with samples with high target values. (b) Solution found by UNCOVER looking for association with low target values. (c) Solution found by UNCOVER when the target values are ignored. Each panel shows the value of the target (top row) for various samples (columns), with yellow being negative and blue being positive values. For each gene in the solution, alterations in each sample are shown in dark blue, while samples not altered are in yellow. The last row shows the alteration profile of the entire solution.

We ran UNCOVER on the GDSC dataset for *k* = 3 and compared the results obtained when the target values are not considered in the analysis, obtained running UNCOVER ILP with *k* = 3 while setting the target values to 1 for all the samples considered in the analysis of a target (S1 Table). The latter analysis corresponds to the extraction of sets with high mutual exclusivity (e.g., by [34]). As expected, the solutions obtained in the two cases are very different: the solution obtained without considering the target values has one alteration in common with the solution obtained by UNCOVER using either positive or negative values of the target for only 11 targets of the 265 in the GDSC dataset, and for no target the solutions share more than 1 alteration. An example of the solutions obtained target using UNCOVER and without considering the target values are shown in Fig 3. We observe that while the solutions obtained considering the target values display an association with the target profile (positive or negative), the solution obtained when the target values are not considered, while covering a large set of samples, does not display any positive or negative association with the target profile. To asses the association between target values and alterations more consistently we calculated the point biserial coefficient [40] for all 265 solutions. The coefficient varies between −1 and +1 with 0 implying no correlation. The average value obtained when ignoring the target is −0.02 with standard deviation 0.05, while the the average value obtained by UNCOVER is 0.20 with standard deviation 0.05. These results show that a mutual exclusivity analysis that disregards the values of the target does not identify sets of mutually exclusive alterations associated with target values. In addition, the genes in solution identified by considering the drug target have a much more significant enrichment in known cancer genes, as reported in [11], than the genes in solution identified disregarding the values of the target (*p* = 3 × 10^−12^ vs *p* = 10^−2^).

### Comparison with REVEALER

**Fig 4:**
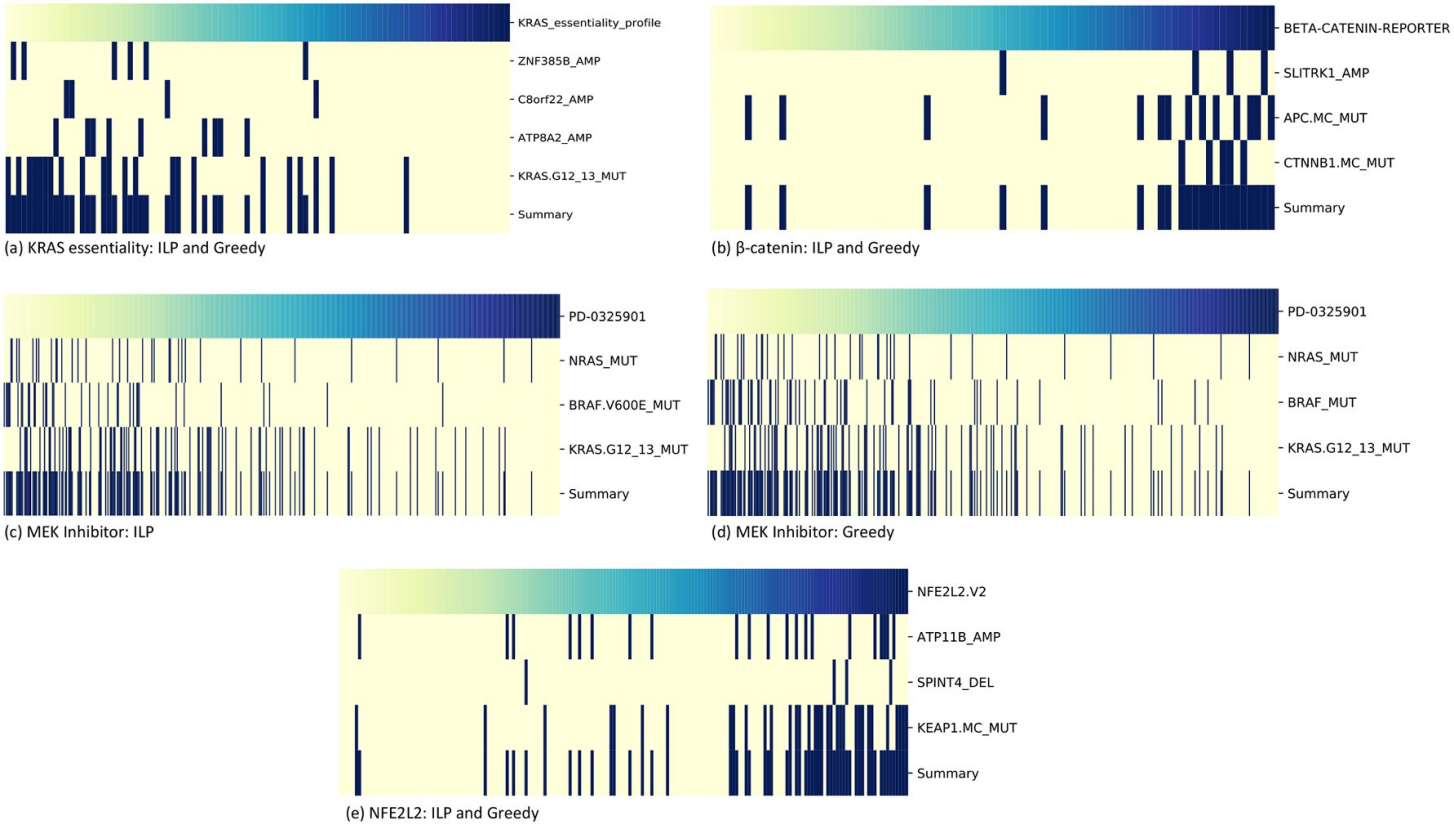
Results of UNCOVER on four cancer datasets from [33]. (a) Solution found by ILP and greedy for KRAS essentiality target. (b) Solution found by ILP and greedy for *β*-catenin activation target. (c) Solution found by ILP for MEK inhibitor target. (d) Solution found by greedy for MEK inhibitor target. (e) Solution found by ILP and greedy for NFE2L2 activation target. Each panel shows the value of the target (top row) for various samples (columns), with yellow being negative and blue being positive values. For each gene in the solution, alterations in each sample are shown in dark blue, while samples not altered are in yellow. The last row shows the alteration profile of the entire solution.

We run the greedy algorithm and the ILP from UNCOVER on the same four datasets considered by the REVEALER publication [33]. We used the same values of *k* used in [33], that is *k* = 3 for all the datasets, except from the KRAS dataset where *k* = 4 was used. For each dataset we recorded the solution reported by the greedy algorithm, the solution reported by the ILP, the value of the objective functions for such solutions and the running time to obtain such solutions. For ILP solutions, we also performed the permutation test (see Materials and methods) to compute a *p*-value using 1000 permutations. The results are reported in Table 1, in which we also show the results from REVEALER (without initial seeds). Fig 4 shows alteration matrices and the association with the target for the solutions identified by UNCOVER.

**Table 1:** Comparison of UNCOVER with REVEALER on REVEALER’s datasets.

	NFE2L2 activation	MEK-inhibitor	KRAS essentiality	*β*-catenin activation
**UNCOVER(ILP)** **solution**	KEAP1.MC MUT	BRAF.V600E MUT	KRAS.G12 13 MUT	APC.MC MUT
	ATP11B AMP	KRAS.G12 13 MUT	ZNF385B AMP	CTNNB1.MC MUT
	SPINT4 DEL	NRAS MUT	ATP8A2 AMP	SLITRK1 AMP
			C8orf22 AMP	
**Objective value**	46.17	108.32	28.00	22.97
**IC score**	0.58	0.49	0.63	0.67
**p-value**	0.000999	0.000999	0.025974	0.1068931
**Running time (s)**	14	39	9	9
**UNCOVER(Greedy)** **solution**	KEAP1.MC MUT	BRAF MUT	KRAS.G12 13 MUT	APC.MC MUT
	ATP11B AMP	KRAS.G12 13 MUT	ZNF385B AMP	CTNNB1.MC MUT
	SPINT4 DEL	NRAS MUT	ATP8A2 AMP	SLITRK1 AMP
			C8orf22 AMP	
**Objective value**	46.17	104.29	28.00	22.97
**IC score**	0.58	0.5	0.63	0.67
**Running time (s)**	15	35	9	8
**REVEALER** **solution**	KEAP1.MC MUT	BRAF MUT	KRAS.G12 13 MUT	APC.MC MUT
	LRP1B DEL	KRAS.G12 13 MUT	ZNF385B AMP	CTNNB1.MC MUT
	OR4F13P AMP	NRAS MUT	LINC00340 DEL	ITGBL1 AMP
			NUP153 MUT	
**Objective value**	30.35	104.29	21.86	22.12
**IC score**	0.54	0.5	0.6	0.7
**Running time (s)**	1615	4965	1243	787

For each of the four targets (NFE2L2 activation, MEK-inhibitor, KRAS essentiality, *β*-catenin activation) considered in [33], the set of alterations of cardinality *k* reported by our ILP algorithm, by our greedy algorithm, and by REVEALER (without seeds) is reported. *k* is chosen as in [33]. For each pair (algorithm, target) we also report the (objective) value of our objective function for the solution, the value of the IC score (that is, the objective function used in [33]), and the running time of the algorithm for the target. For solutions found by our ILP we also report the *p*-value computed by permutation test using 1000 permutations.

We can see that the greedy algorithm identifies the same solution of the ILP based algorithm in three out of four cases, and that the runtime of the ILP and the runtime of greedy algorithm are comparable and very low (< 40 seconds) in all cases. In contrast, the running time of REVEALER is much higher (> 1000 seconds in most cases). (We included all preprocessing in the reported UNCOVER runtimes in Table 1 to ensure a fair comparison with REVEALER; not including preprocessing our running times are all under 10 seconds). Comparing the alteration matrices of the solutions by UNCOVER and the ones of solutions by REVEALER (S1 Fig) we note that alterations in solutions by UNCOVER tend to have higher mutual exclusivity and to be more concentrated in high weight samples than alterations in solutions by REVEALER. As expected, the value of the objective function we use is much lower for solutions from REVEALER than for solutions from our algorithm.

We then compared the solutions obtained by our algorithms with the solutions from REVEALER in terms of the *information coefficient* (IC), that is the target association score used in [33] as a quality of the solution. Surprisingly, in two out of four datasets UNCOVER, which does not consider the IC score, identifies solutions with IC score *higher* (by at least 5%) than the solutions reported by REVEALER. For the other two cases, in one dataset the IC score is very similar (0.50 vs 0.49) while in the other case the IC score by REVEALER is higher (0.7 vs 0.67) but the solution reported by REVEALER differs from the solution reported by UNCOVER by 1 gene only. Interestingly, the latter is the only case where the solution from the ILP has a *p*-value > 0.1 (*p* < 0.03 in all other cases), and therefore the solutions (by our methods and by REVEALER) for such dataset may be, at least in part, due to random fluctuations of the data.

In terms of biological significance, in most cases the solutions by UNCOVER and by REVEALER are very similar, with cancer relevant genes identified by both methods. For NFE2L2 activation, both methods identify KEAP1, a repressor of NFE2L2 activation [41]. For MEK-inhibitor, both methods find BRAF, KRAS, and NRAS, three well known oncogenic activators of the MAPK signaling pathway, which contains MEK as well. For KRAS essentiality, both methods report mutations in KRAS in the solution. For *β*-catenin activation, both methods identify CTNNB1 mutations and APC mutations, that is known to be associated to *β*-catenin activation [42]. These results show that UNCOVER identifies relevant biological solutions that are better than the ones identified by REVEALER when evaluated using our objective function *and* also when evaluated according to the objective function of REVEALER with a running time that is on average two orders of magnitude smaller than required by REVEALER. Since UNCOVER and REVEALER consider two different objective functions, it is unclear whether the improvement in running time comes from differences in implementation choices or from a inherently different computational complexity. However, since UNCOVER’s objective function is easier to compute than REVEALER’s objective function, we believe that the use of our objective function plays an important role in the efficiency of UNCOVER.

We also compared the solutions obtained by UNCOVER and by REVEALER on the GDSC dataset (S2 Table). For both algorithms we obtained the solutions for *k* = 3. For UNCOVER, we considered the solution returned by the ILP. For REVEALER, we could only obtain solutions for 246 targets, since for the other targets REVEALER terminated with an error message. Due to the high running time of REVEALER, we only obtained sets of alterations associated with positive values of the target (Table 2). For 33 targets the solution by UNCOVER and the solution by REVEALER share 1 alteration, while for 33 targets the solution by UNCOVER and the solution by REVEALER share 2 alterations; for no target UNCOVER and REVEALER report the same solution. This shows that the two methods identify completely different solution in most (> 73%) of the cases. We compared the solutions obtained by UNCOVER and by REVEALER using the IC score considered by REVEALER but not from UNCOVER: surprisingly, in more than 50% of the cases (113 out of 208) the IC score of the solution from UNCOVER is higher than the IC of the solution from REVEALER. On the other hand, for all targets the solution by REVEALER is worst than the solution by UNCOVER when the UNCOVER objective function is considered. We also compared UNCOVER and REVEALER evaluating the association between target values and alterations in the solutions using a measure of association that is not considered by the two algorithms. In particular, we considered the point biserial correlation coefficient [40]. In more than 95% of the cases (199 out of 208) the point biserial correlation coefficient between the solution from UNCOVER and the target is higher than the point biserial correlation coefficient between the solution from REVEALER and the target, that is, the solution from UNCOVER has an higher association with the target than the solution from REVEALER. On average, the solution from UNCOVER has a point biserial correlation coefficient that is 37% higher than the point biserial correlation coefficient of the solution from REVEALER. Moreover, the average effect size of solutions from UNCOVER is more than 80% higher than the average effct size of solutions from REVEALER (Table 2). In addition, the genes in solutions from UNCOVER have a much higher enrichment (*p* = 3 × 10^−13^; 7-fold enrichment) for known cancer genes than solutions from REVEALER (*p* = 2 × 10^−4^; 3-fold enrichment). Analogously, more KEGG pathways display a significant enrichment in genes from UNCOVER solutions than from REVEALER solutions (22 vs 11). We also compared the running time of the two methods: UNCOVER required 3 hours to complete the analysis, while REVEALER required 9 days. Overall, these results show that UNCOVER obtains better results than REVEALER not only in terms of the UNCOVER objective function but also in terms of the score from REVEALER as well as in terms of a independent measure of association, while being 70 times faster than REVEALER.

**Table 2:** Comparison of UNCOVER with REVEALER on GDSC dataset.

	Number of genes	Avg. effect size	Cancer genes enrichment *p*-value (fold enrich.)	Enriched KEGG pathways
REVEALER	570	0.11	2 × 10^−4^ (3)	11
UNCOVER	491	0.20	3 × 10^−12^ (7)	22

For each algorithm we report the distinct number of genes in its solutions, the average effect size of the algorithm’s solutions, the *p*-value and fold enrichment for known cancer genes, and the number of KEGG pathways enriched for genes in the solutions by the algorithm.

### Results on simulated data

For each combination we generated 10 simulated datasets as described in Materials and methods. Each dataset contains a *planted* set of 5 alterations associated with the target. We used both the greedy algorithm and the ILP from UNCOVER with *k* = 5 to attempt to find the 5 correct alteration, and evaluated our algorithms both in terms of fraction of the correct (i.e., planted) solution reported and running time.

**Fig 5:**
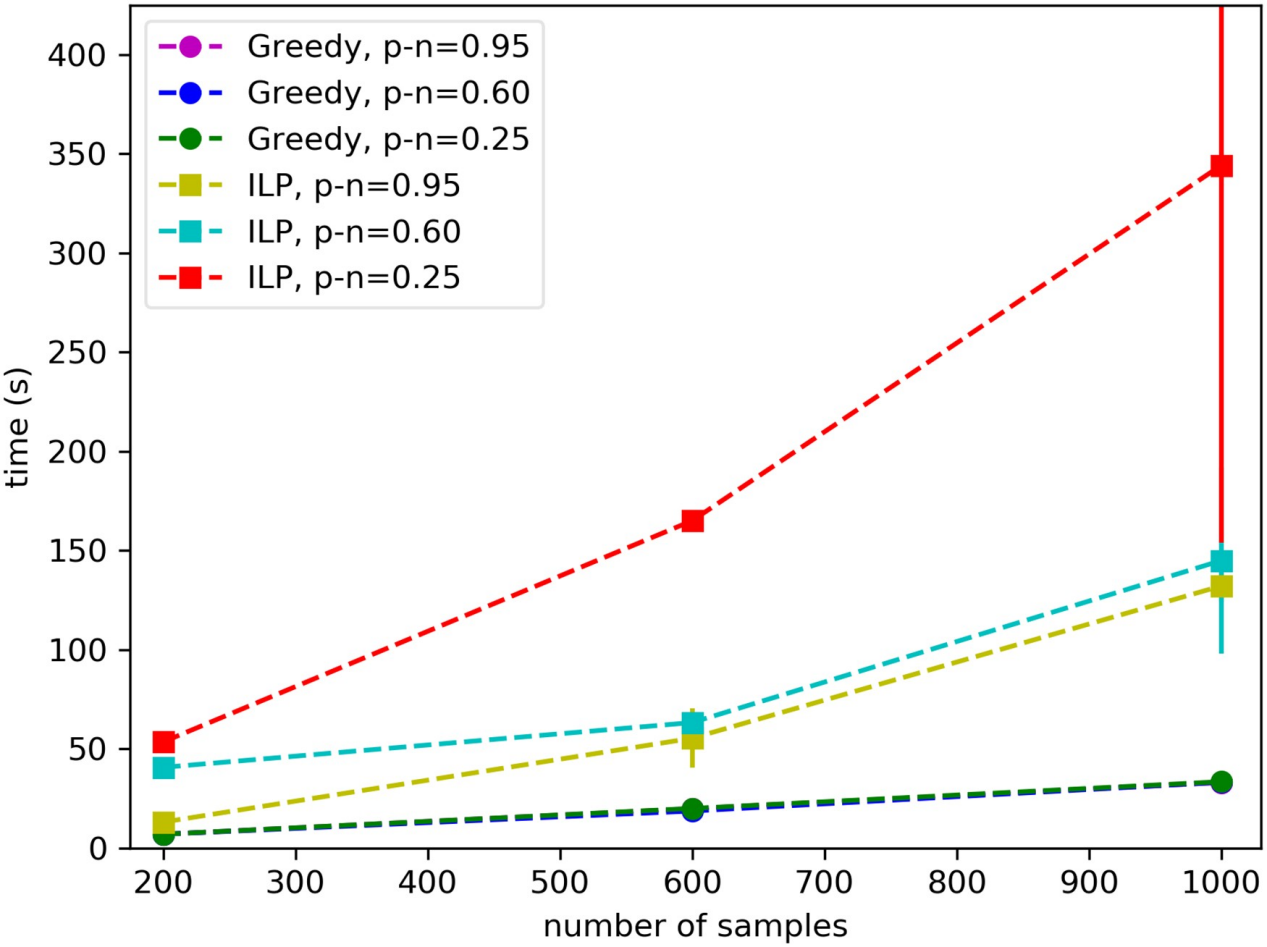
Running time of UNCOVER on simulated data. The running time (expectation and standard deviation) of the greedy algorithm and of the ILP approach are shown for different number of samples and the difference *p* − *n* between the fraction *p* of samples with positive target and the fraction *n* of samples with negative target covered by the the correct solution.

As shown in Fig 5, the greedy algorithm is faster than the ILP for all datasets, and the difference in running time increases as the number *m* of samples increases, with the runtime of the greedy algorithm being almost two orders of magnitude smaller than the runtime of the ILP for *m* = 1000 samples. In addition, for a fixed number of samples and alterations, the running time of the greedy algorithm is constant, that is it does not depend on the properties of the planted solution, while the running time of the ILP varies greatly depending on these parameters. For *m* = 10, 000 samples the running time of the ILP becomes extremely high, so we restricted to consider only two sets of parameters (*p* − *n* = 0.95 and *p* − *n* = 0.2). In this case the ILP took between 44 minutes and 7 hours to complete, while the greedy algorithm terminates in 5 minutes.

**Fig 6:**
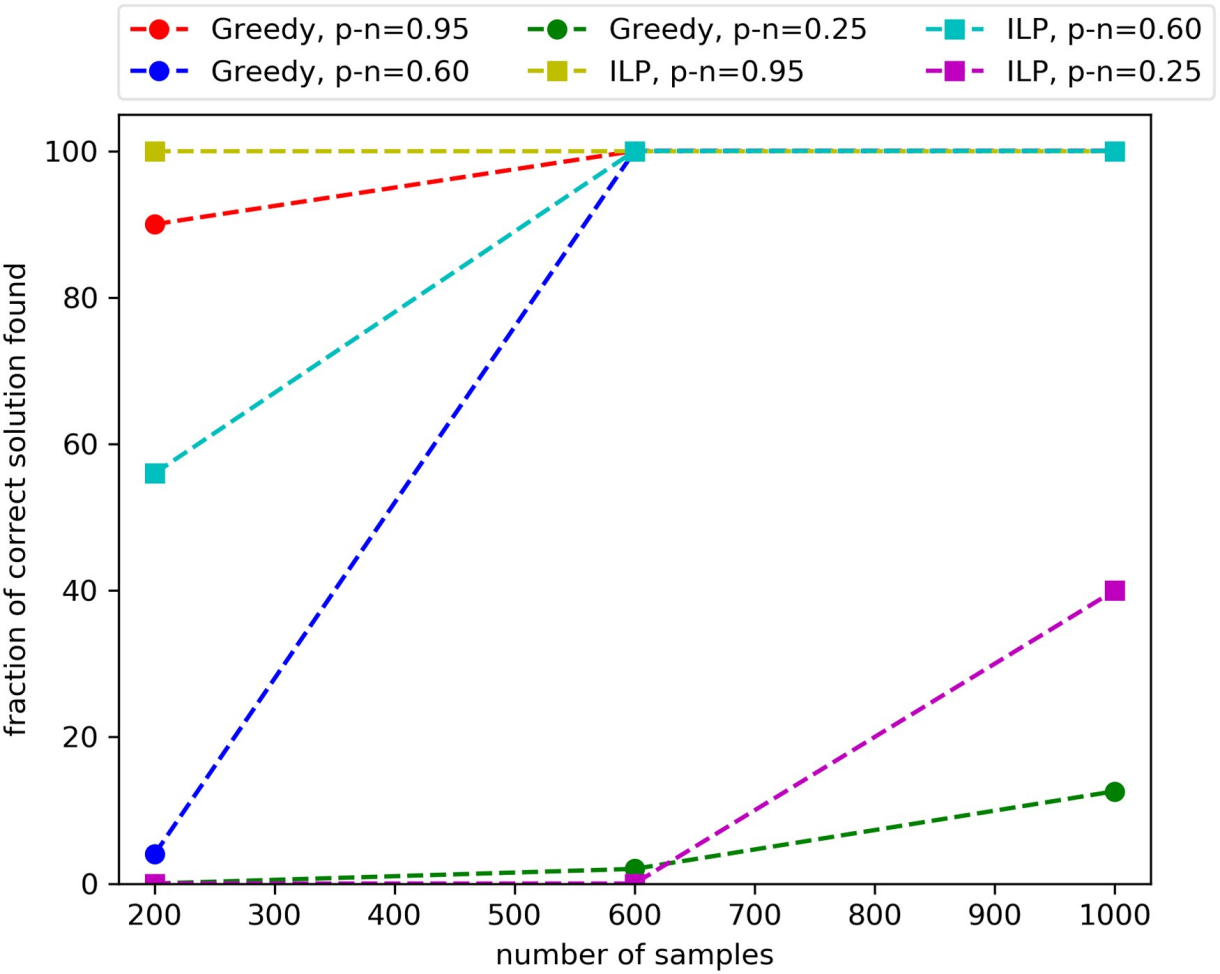
Quality of solutions of UNCOVER on simulated data. The fraction of genes in the planted (i.e., correct) solution found by the greedy algorithm and by the ILP approach are shown for different number of samples and the difference *p* − *n* between the fraction *p* of samples with positive target and the fraction *n* of samples with negative target covered by the the correct solution.

In terms of the quality of the solutions found, as expected the ILP outperforms the greedy (Fig 6) but the difference among the two tends to disappear when the number of samples is higher. In addition, since the ILP finds the optimal solution, we can see that for a limited number of samples we may not reliably identify the planted solution with 200 samples unless the planted solution appears almost only in positive targets and in almost all of them (*p* − *n* = 0.95), while for m = 1000 we can reliably identify the planted solution using both the ILP and the greedy algorithm even when the association with the target is weaker (*p* − *n* = 0.6). When *m* = 10, 000, both the ILP and the greedy algorithm perform well in terms of the quality of the solution: the ILP finds the correct alterations on every experiment and the greedy identifies the whole planted solution in all experiments but one for *p* − *n* = 0.2, for which it still reports a solution containing 4 out of 5 genes in the planted solution.

These results show that for a large number of samples the greedy algorithm reliably identifies sets of alterations associated with the target, as predicted by our theoretical analysis, and is much faster than the ILP. For smaller sample size the ILP identifies better solutions than the greedy and has a reasonable running time.

### Analysis of Achilles project data

The efficiency of UNCOVER renders the analysis of a large number of targets, such as the ones available through the Achilles project, possible. After preprocessing the dataset included 5690 functional phenotypes as targets, and for each of these the CCLE provides alteration information for 205 samples and 31137 alterations. In total we have therefore run 11380 instances (i.e., 5690 targets screened for positive and for negative associations) looking for both positive and negative association with target values. Since the number of samples (205) is relatively small, we have run only the ILP from UNCOVER on the whole Achilles dataset and looked for solutions with *k* = 3 genes. The runtime of UNCOVER to find both positive and negative associations, including preprocessing, is 24 hours. Based on the runtime required on the instances reported in [33] (see the Section Comparison with REVEALER), running REVEALER on this dataset would have required about 5 months of compute time.

To identify statistically significant associations with targets in the Achilles project dataset we used a nested permutation test. We first run the permutation test with 10 permutations on all instances (i.e., on all targets for both positive association and negative association). We then considered all the instances with the lowest p-value (1/11) and performed a permutation test with 100 permutations only for such instances. We the iterated such procedure once more, selecting all the instances with lowest p-value (1/101) and performing a permutation test with 1000 permutations only for such instances. For positive association we found 60 solutions with *p*-value < 0.001, and for negative association we found 102 solutions with *p*-value < 0.001. The solutions with *p*-value < 0.001 (with 1000 permutations) are reported in S3 Table. See S2 Fig for some corresponding alteration matrices.

**Fig 7:**
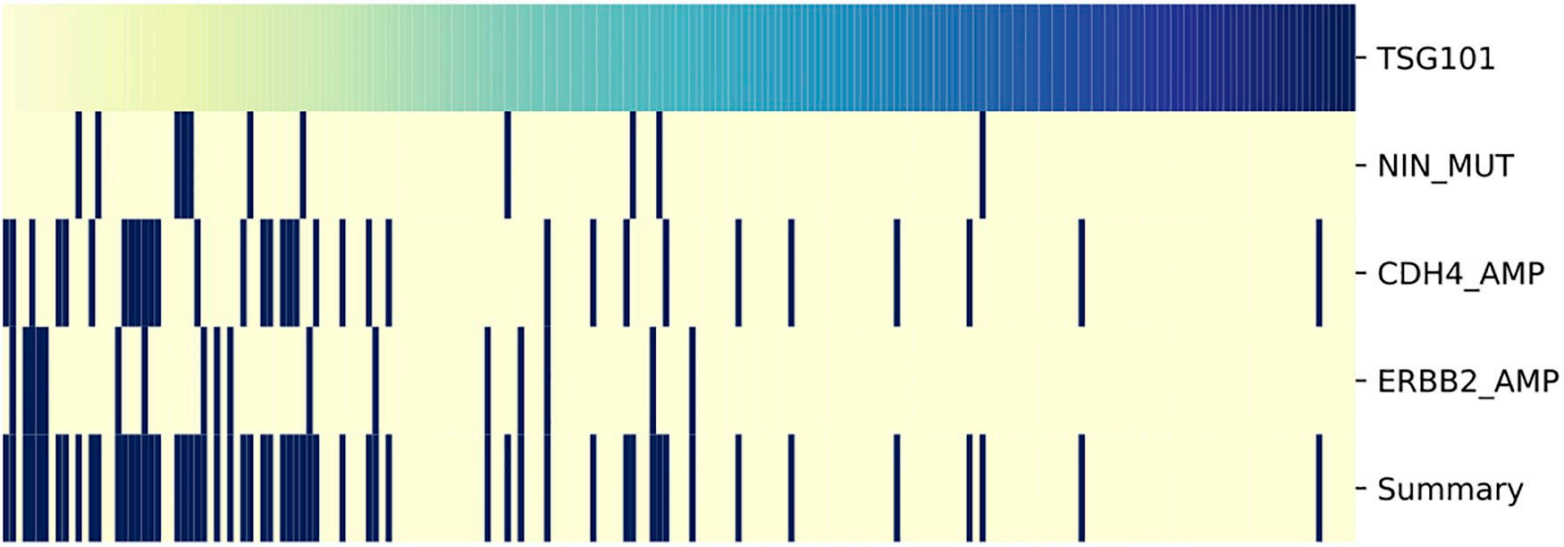
Solution by UNCOVER for silencing of TSG101 (data from Achilles project). The alteration matrix of genes in the solution identified by UNCOVER as associated to reduced cell viability is reported. The value of the target (top row) for various samples (columns) is shown, with yellow being negative and blue being positive values. For each gene in the solution, alterations in each sample are shown in dark blue, while samples not altered are in yellow. The last row shows the alteration profile of the entire solution.

The genes in the solutions by UNCOVER with p-value 1/1001 are enriched (*p* = 2 × 10^−12^ by Fisher exact test; 8 fold enrichment) for well-known cancer genes. We also tested whether genes in solutions by UNCOVER (with p-value 1/1001) are enriched for interactions, by comparing the number of interactions in iRefIndex [43] among genes in such solution with the number of interactions in random sets of genes of the same cardinality. Genes in solutions by UNCOVER are significantly enriched in interactions (*p* = 7 × 10^−3^ by permutation test; 2 fold enrichment). In addition, the genes in solutions by UNCOVER are also enriched in genes in well-known pathways: 12 KEGG pathways [44] have a significant (corrected *p* ≤ 0.05) overlap with genes in solutions by UNCOVER and four of these (endometrial cancer, glioma, hepatocellular carcinoma, EGFR tyrosine kinase inhibitor resistance) are cancer related pathways. In addition, the *targets* (i.e., genes) with solutions of *p*-value 1/1001 are enriched (*p* = 5 × 10^−4^ by permutation test; 6 fold enrichment) for interactions in iRefIndex and for well-known cancer genes (*p* = 2 × 10^−12^ by Fisher exact test; 8 fold enrichment) as reported in [11]. These results show that UNCOVER enables the identification of groups of well known cancer genes with significant associations to important targets in large datasets of functional target profiles. For example, for target (i.e., silenced gene) TSG101, related to cell growth, UNCOVER identifies the gene set shown in Fig 7 as associated to reduced cell viability. ERBB2 is a well known cancer gene and CDH4 is frequently mutated in several cancer types, and both are associated to cell growth.

### Analysis of GDSC project data

We use UNCOVER to analyze the GDSC project data, identifying sets of alterations associated with drug sensitivity. After preprocessing, the dataset included 64144 alterations and 265 targets, and for each of these the number of cell lines with available data varied between 240 and 705. In total we have therefore run 530 instances (i.e., 265 targets screened for positive and for negative associations) looking for both positive and negative association with target values.

We used the UNCOVER ILP for all instances to obtain solutions with *k* = 3 genes. For each solution, we use 100 permutations to compute its *p*-value. For positive association we found 51 solutions with *p*-value < 0.01, and for negative association we found 41 solutions with *p*-value < 0.01. We used the following procedure to focus on the most significant solutions: we run UNCOVER with *k* = 4 and computed the *p*-values for the solutions using 100 permutations; we then identified targets whose solution for *k* = 3 have *p*-value < 0.01 and are contained in the solution for the same target with *k* = 4 and have *p*-value *p* < 0.01 for *k* = 4. In total, this procedure identifies 23 solutions for positive association and 22 solutions for negative associations. These solutions are reported in S4 Table.

The genes in the solutions identified as above are enriched (*p* = 9 × 10^−10^ by Fisher exact test; 20 fold enrichment) for well-known cancer genes, as reported in [11]. We also tested whether these genes in solutions are enriched for interactions, by comparing the number of interactions in iRefIndex [43] among genes in such solution with the number of interactions in random sets of genes of the same cardinality. Genes in solutions by UNCOVER are significantly enriched in interactions (*p* = 2 × 10^−2^ by permutation test; 6 fold enrichment). In addition, these genes are also enriched in genes in well-known pathways: 21 KEGG pathways [44] have a significant (corrected *p* ≤ 0.05) overlap with genes in solutions by UNCOVER and 19 of these are cancer related pathways (e.g., ErbB signaling pathway) or related to drug resistance (e.g., EGFR tyrosine kinase inhibitor resistance).

**Fig 8:**
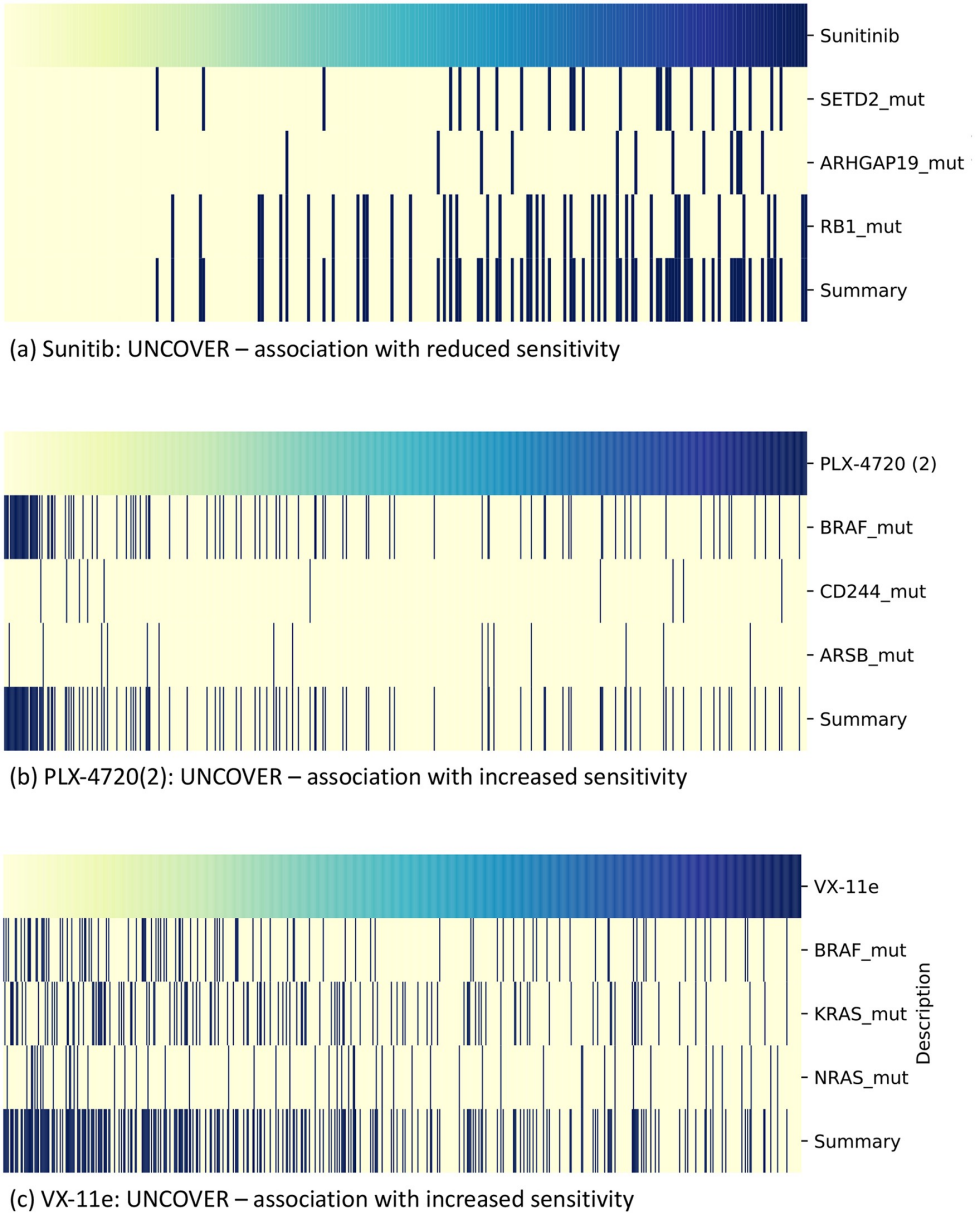
Solution by UNCOVER on GDSC drug sensitivity data data. The alteration matrix of genes in some solutions identified by UNCOVER as associated to drug sensitivity for different targets. (a) Solution for reduced sensitivity to Sunitinib. (b) Solution for increased sensitivity to PLX-4720-2. (c) Solution for increased sensitivity to VX-11e. Each panel shows the value of the target (top row) for various samples (columns), with yellow being negative and blue being positive values. For each gene in the solution, alterations in each sample are shown in dark blue, while samples not altered are in yellow. The last row shows the alteration profile of the entire solution.

For Palbociclib, UNCOVER identifies RB1 mutations, GRB7 amplifications, and RB1 deletions with significant association with reduced sensitivity to drug. RB1 is a well known cancer gene. The alterations are shown in Fig 3a. While RB1 mutations and RB1 deletions are significantly associated when considered in isolation (the association of single alterations with drug sensitivity and the drug targets have been obtained from https://www.cancerrxgene.org/), GRB7 amplification is not associated with the target values when considered in isolation. GRB7 encodes a growth factor receptor-binding protein that interacts with epidermal growth factor receptor (EGFR). Both RB1 and EGFR are related to the cell cycle pathway, that is the pathway target of the compound, and the drug targets (CDK4, CDK6) as well EGFR are members of the PI3K-AKT pathway. For Sunitinib, UNCOVER identifies mutations in SETD2, ARHGAP19, and RB1, with significant association with reduced sensitivity to drug. The alterations are shown in Fig 8a. RB1 is a well known cancer gene and SETD2 has tumor suppressor functionality. None of these alterations have significant association with drug sensitivity when considered in isolations. RB1 and SETD2 are involved in protein localization to chromatin, and ARHGAP19 is part of Rho mediated remodeling. For PLX-4720-2, UNCOVER identifies mutations in BRAF, CD244, and ARSB with significant association to increased sensitivity to drug. The alterations are shown in Fig 8b. BRAF is a well-known cancer gene; it is the target of the compound and BRAF mutations have significant association to increased sensitivity to the compound, while the other two alterations do not. BRAF and CD244 are part of natural killer cell mediated cytotoxicity pathway, while ARSB is involved in the regulation of cell adhesion, cell migration and invasion in colonic epithelium [45], and is also part of metabolism related pathways. For VX-11e, UNCOVER identifies mutations in BRAF, KRAS, and NRAS, with significant association to increased sensitivity to drug. The alterations are shown in Fig 8c. Only BRAF mutations have significant association with the target when considered in isolation. The pathway target for the compound is the ERK MAPK signaling pathway, to which all three alterations are related. All three genes have well identified roles in cancer. These results show that UNCOVER enables the identification of groups of relevant genes, many related to cancer, with significant associations to important targets in large datasets of drug sensitivity profiles.

### Conclusion

In this work we study the problem of identifying sets of mutually exclusive alterations associated with a quantitative target profile.

We provide a combinatorial formulation for the problem, proving that the corresponding computational problem is NP-hard. We design two efficient algorithms, a greedy algorithm and an ILP-based algorithm, for the identification of sets of mutually exclusive alterations associated with a target profile. We provide a formal analysis for our greedy algorithm, proving that it returns solutions with rigorous guarantees in the worst-case as well under a reasonable generative model for the data. We implemented our algorithms in our method UNCOVER, and showed that it finds sets of alterations with a significant association with target profiles in a variety of scenarios. By comparing the results of UNCOVER with the results of REVEALER on four target profiles used in the REVEALER publication [33] and on a large dataset from the GDSC project, we show that UNCOVER identifies better solutions than REVEALER, even when evaluated using REVEALER objective function. Moreover, UNCOVER is much faster than REVEALER, allowing the analysis of large datasets such as the dataset from project Achilles and from the GDSC project, in which UNCOVER identifies a number of associations between functional target profiles and gene set alterations.

Our tool UNCOVER (as well as REVEALER) relies on the assumption that the mutual exclusivity among alterations is due to functional complementarity. Another explanation for mutual exclusivity is the fact that each cancer may comprise different subtypes, with different subtypes being characterized by different alterations [27]. UNCOVER can be used to identify sets of mutually exclusive alterations associated with a specific subtype whenever the subtype information is available, by assigning high weight to samples of the subtype of interest and low weight to samples of the other subtypes. In addition, while we consider a penalty based on mutual exclusivity, other types of penalties may be used to identify sets of alterations associated with a target profile. The study of the theoretical properties of the problem and the analysis of the results with different penalties are interesting directions of future research.

## Supporting information

S1 Fig Results of UNCOVER and REVEALER on four cancer datasets from [33]. (a) Solution found by ILP and greedy for KRAS essentiality target. (b) Solution found by ILP and greedy for *β*-catenin activation target. (c) Solution found by ILP for MEK inhibitor target. (d) Solution found by greedy for MEK inhibitor target. (e) Solution found by ILP and greedy for NFE2L2 activation target. The value of the target (top row) for various samples (columns) is shown, with yellow being negative and blue being positive values. For each gene in the solution, alterations in each sample are shown in dark blue, while samples not altered are in yellow. The last row shows the alteration profile of the entire solution.
(PDF)

S2 Fig Alteration matrices for some results of UNCOVER the Achilles project data. The alteration matrix of genes in some solutions identified by UNCOVER as associated to increased cell viability for different targets. (a) ACSL3 (b) HNRNPH3 (c) MAP3K1 (d) MGAT4C. Each panel shows the value of the target (top row) for various samples (columns), with yellow being negative and blue being positive values. For each gene in the solution, alterations in each sample are shown in dark blue, while samples not altered are in yellow. The last row shows the alteration profile of the entire solution.
(PDF)

S1 Appendix Supplementary text. Proofs of Proposition 2 and of Proposition 3.
(PDF)

S1 Table Solutions found by UNCOVER on the GDSC dataset when the target values are ignored. The table reports the solutions each target, the objective function value, and the point biserial correlation coefficient.
(XLSX)

S2 Table Solutions found by UNCOVER and by REVEALER on the GDSC dataset. The table reports the solutions associated to positive values of the target. For each target and algorithm we report the solution, the objective function value, the IC score, the point biserial correlation coefficient and its *p*-value. The number of alterations shared by UNCOVER’s solution and REVEALER’s solutions are shown as well.
(XLSX)

S3 Table Solutions found by UNCOVER on the Achilles dataset. The table reports the solutions associated to negative values of the target and the solutions associated to positive values of the target. In both cases only solutions with the lowest *p*-value are reported. For each target we report the objective function value for the optimal solution, the set of alterations of cardinality 3 and the p-value computed by permutation test using 1000 permutations.
(XLSX)

S4 Table Solutions found by UNCOVER on the GDSC dataset. The table reports the solutions associated to negative values of the target and the solutions associated to positive values of the target. In both cases only solutions filtered using the procedure described in Section “Analysis of GDSC project data”.
(XLSX)
