# Supplementary material for: Efficient algorithms to discover alterations with complementary functional association in cancer
Source: PLoS Comput Biol. 2019 May 23;15(5):e1006802. doi: 10.1371/journal.pcbi.1006802 (PMC6550413; doi:10.1371/journal.pcbi.1006802)
Supplement: S1 Fig — (a) Solution found by ILP and greedy for KRAS essentiality target. (b) Solution found by ILP and greedy for β-catenin activation target. (c) Solution found by ILP for MEK inhibitor target. (d) Solution found by greedy for MEK inhibitor target. (e) Solution found by ILP and greedy for NFE2L2 activation target. The value of the target (top row) for various samples (columns) is shown, with yellow being negative and blue being positive values. For each gene in the solution, alterations in each sample are shown in dark blue, while samples not altered are in yellow. The last row shows the alteration profile of the entire solution. (PDF) [file pcbi.1006802.s001.pdf]

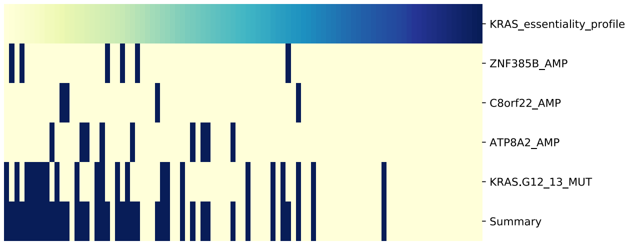

(a) KRAS essentiality: UNCOVER

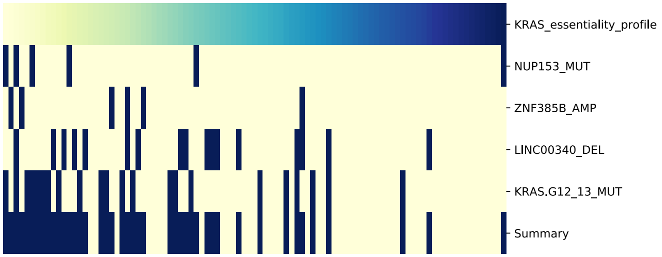

(b) KRAS essentiality: REVEALER

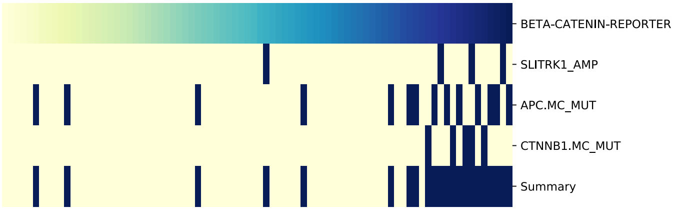

(c) Beta-catenin: UNCOVER

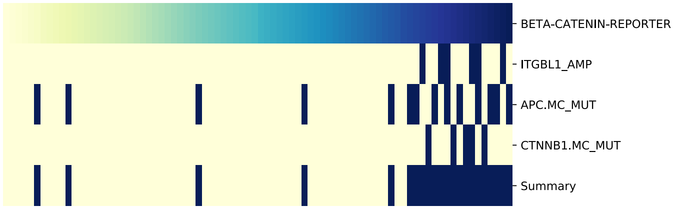

(d) Beta-catenin: REVEALER

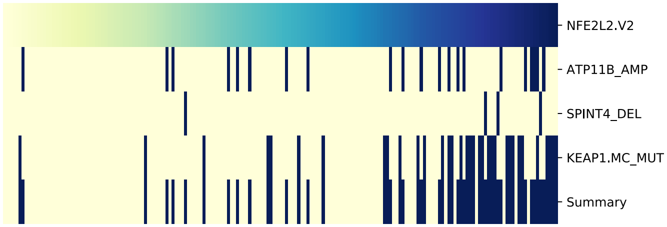

(e) NFE2L2: UNCOVER

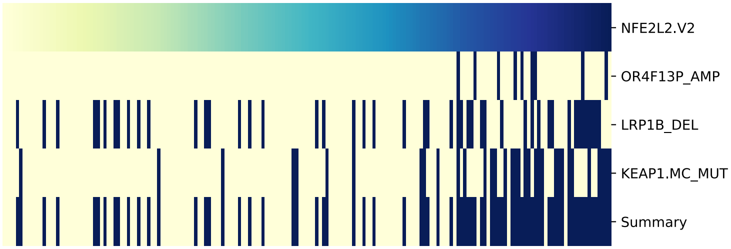

(f) NFE2L2: REVEALER

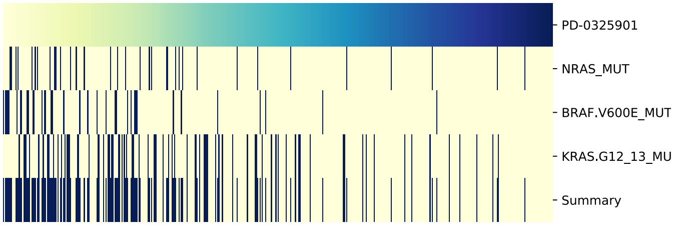

(g) MEK Inhibitor: UNCOVER (ILP)

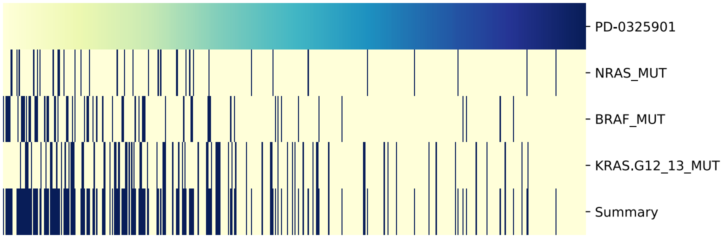

(h) MEK Inhibitor: REVEALER

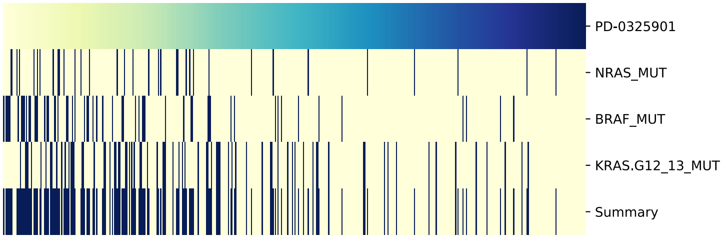

(i) MEK Inhibitor: UNCOVER (Greedy)
