# Supplementary figures and images for: Efficient algorithms to discover alterations with complementary functional association in cancer
Source: PLoS Comput Biol. 2019 May 23;15(5):e1006802. doi: 10.1371/journal.pcbi.1006802 (PMC6550413; doi:10.1371/journal.pcbi.1006802)

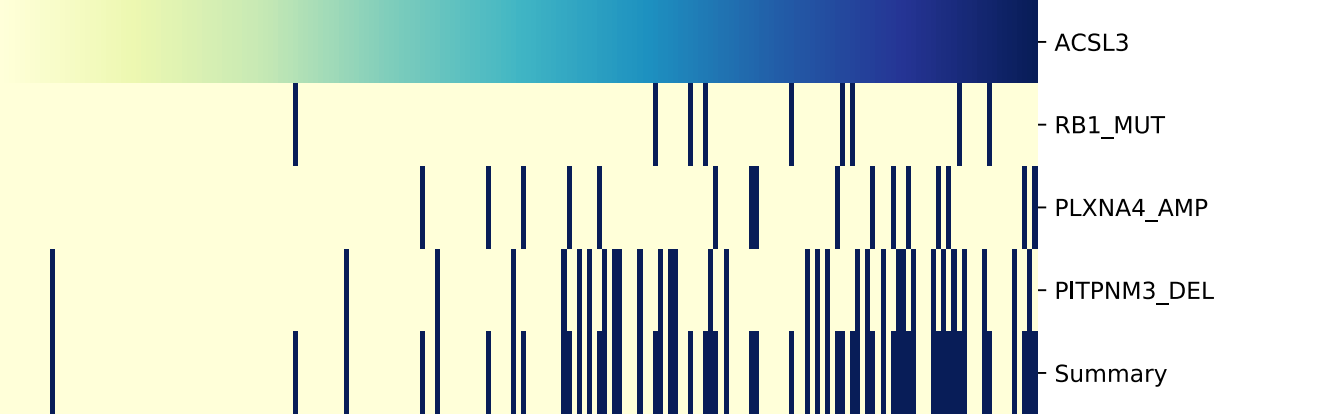

(a) ACSL3

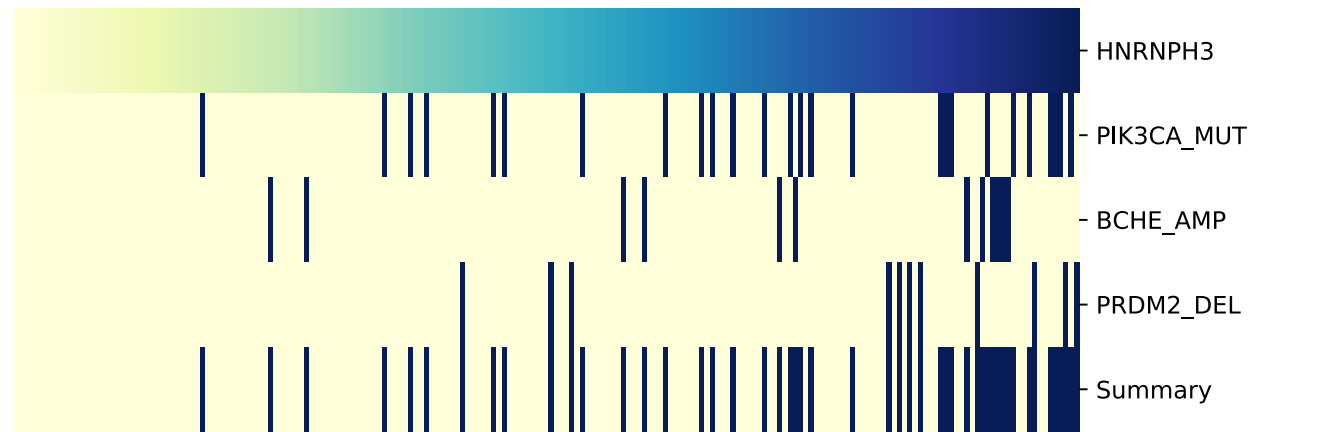

(b) HNRNPH3

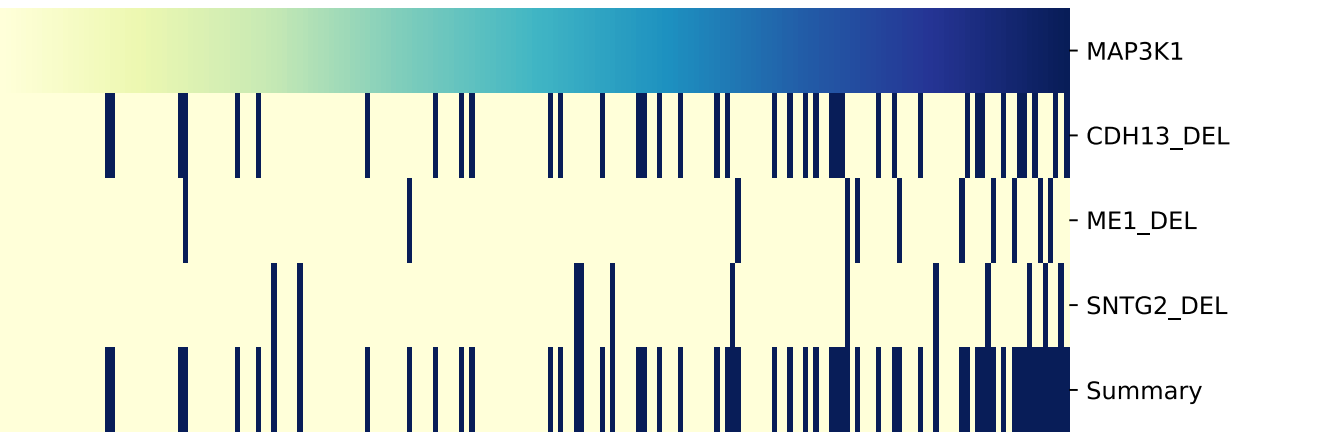

(c) MAP3K1

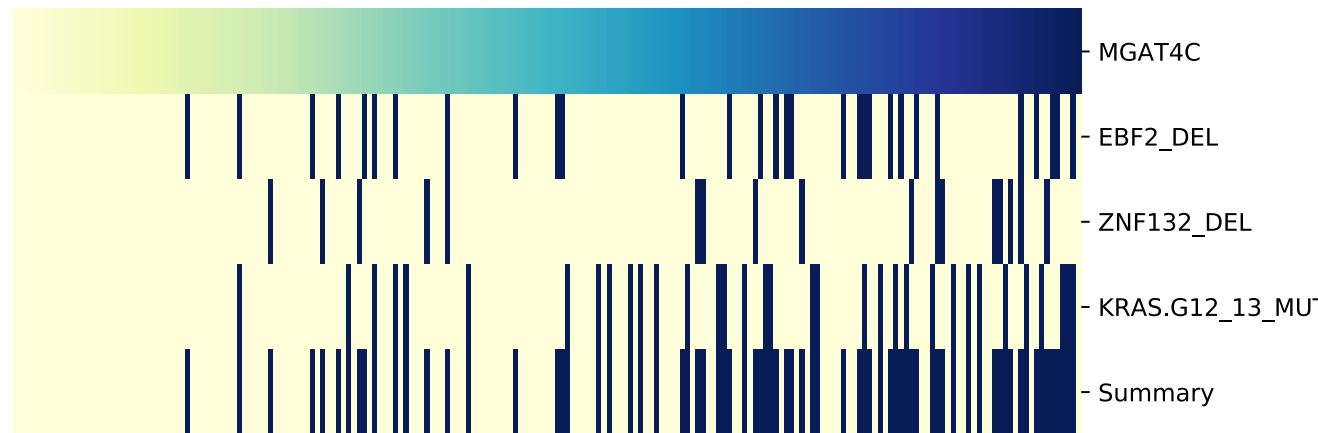

(d) MGAT4C

Supplement: S2 Fig — The alteration matrix of genes in some solutions identified by UNCOVER as associated to increased cell viability for different targets. (a) ACSL3 (b) HNRNPH3 (c) MAP3K1 (d) MGAT4C. Each panel shows the value of the target (top row) for various samples (columns), with yellow being negative and blue being positive values. For each gene in the solution, alterations in each sample are shown in dark blue, while samples not altered are in yellow. The last row shows the alteration profile of the entire solution. (PDF) [file pcbi.1006802.s002.pdf]
