# Supplementary material for: Efficient algorithms to discover alterations with complementary functional association in cancer
Source: PLoS Comput Biol. 2019 May 23;15(5):e1006802. doi: 10.1371/journal.pcbi.1006802 (PMC6550413; doi:10.1371/journal.pcbi.1006802)
Supplement: S1 Appendix — Proofs of Proposition 2 and of Proposition 3. (PDF) [file pcbi.1006802.s003.pdf]

Supplementary Text for
Efficient Algorithms to Discover Alterations with Complementary Functional Association in Cancer

Rebecca Sarto Basso[*]
rebeccasarto@berkeley.edu

Dorit S. Hochbaum[*]
hochbaum@ieor.berkeley.edu

Fabio Vandin[†,‡,§,¶]
fabio.vandin@unipd.it

Proposition 1. *There are instances of the Target Associated k-Set such that $W(\hat{S}) = W(S^*)/k$.*

Proof. To see that the bound is tight just consider the following example. We want to pick k sets out of n sets $A_1 ... A_n$. Sets $A_1 ... A_k$ include 2 elements of respective weight $a \geq 0$ and $b = a/(k-1)$. Subset A_{k+1} includes all the elements of weight b from the previous k sets and one element with a small weight ϵ. Each of the remaining sets $A_{k+2} ... A_n$ include an arbitrary number of elements with overall weight ≤ 0. We choose a penalty of value a. Note that one can choose the weights of elements in sets $A_{k+2} ... A_n$ in such a way that the average of all positive normalized weights is equal to a. Clearly the optimal solution to the Target Associated k-Set problem consists of sets $A_1 ... A_k$ with an objective value of $k(a+b)$. The greedy algorithm will pick set A_{k+1} at the first iteration and then assign a new weight to its elements equal to $-a$. The updated weight of sets $A_1 ... A_k$ is now 0 and the algorithm will stop and output A_{k+1} as the solution, giving an approximation ratio of

$$\frac{kb + \epsilon}{k(a+b)} = \frac{1}{k} + \frac{\epsilon}{kb}$$

□

Proposition 2. *If $m \in \Omega\left(k^2 \ln(n/\delta)\right)$ samples from the generative model above are provided to the greedy algorithm, then the solution of the greedy algorithm is H with probability $\geq \delta$.*

Proof. We prove that in iteration i of the greedy algorithm, conditioning on the current solution being a set S with $S \subset H$, then the greedy algorithm adds a gene in $H \setminus S$ to the solution with probability $\geq delta/k$, and that the first gene added by the greedy algorithm is $g \in H$. The result then follows by union bound on the k iterations of the greedy algorithm.

Consider the first iteration of the greedy algorithm and consider a gene $g \in G$. Note that if $g \notin H$ then $\mathbf{E}[W(\{g\})] \leq 0$, since $\mathbf{E}[\sum_{j \in A_g} w_j] = 0$ because the samples in which g is mutated are taken uniformly at random while $\sum_{j \in A_g}(c_S(j) - 1) \geq 0$. If $g \in H$ by the assumptions of the model we have $\mathbf{E}[W(\{g\})] \geq \frac{m}{kc'''}$ for a constant $c''' \geq 1$. Note that $W(\{g\})$ can be written as the sum $\sum_{i=1}^{m} X_i$ of random variables (r.v.'s) X_i where X_i is the contribution of sample i to $W(\{g\})$ with $X_i \in [-1, 1]$. By the Azuma-Hoeffding

[*]Department of Industrial Engineering and Operations Research, University of California at Berkeley (USA).
[†]Department of Information Engineering, University of Padova (Italy).
[‡]Department of Computer Science, Brown University (USA).
[§]Department of Mathematics and Computer Science, University of Southern Denmark (Denmark).
[¶]Corresponding author.

inequality [?] and union bound (on the n genes) the first gene chosen by the greedy algorithm is not gene $g \in H$ with probability $\leq e^{-\frac{2m^2}{4mk^2(c''')^2}}$ which is $\leq \delta/k$ when $m \in \Omega\left(k^2 \ln(nk/\delta)\right)$.

Now assume that in iteration i, for the current solution $S \subset H$. Consider a gene $g \in G \setminus H$, then $\mathbf{E}[W(S \cup \{g\}) - W(S)] \leq 0$, since $\mathbf{E}[\sum_{j \in \cup_{s \in S \cup g} A_s} w_j - \sum_{j \in \cup_{s \in S} A_s} w_j] \leq 0$ (by the assumptions of the model $W(S) > 0$ and the fact that alterations in $\{g\}$ are placed uniformly at random among samples) and $\mathbf{E}[\sum_{j \in \cup_{s \in S \cup g}} (c_S(j) - 1) - \sum_{j \in \cup_{s \in S}} (c_S(j) - 1)] \geq 0$ (because for each sample i, the number of alterations of $S \cup \{g\}$ in i is a superset of the number of alterations of S in i). Consider now a gene $g \in H \setminus S$: by the assumptions of the model $\mathbf{E}[W(S \cup \{g\}) - W(S)] \leq \frac{m}{kc'''}$ for a constant $c''' > 1$. Note that $\mathbf{E}[W(S \cup \{g\}) - W(S)$ can be written as the sum of $\sum_{i=1}^{m} X_i$ of random variables (r.v.'s) X_i where X_i is the contribution of sample i in the increase in weight from $W(S)$ to $W(S \cup \{g\})$, where $X_i \in [-1, 1]$. By the Azuma-Hoeffding inequality and union bound (on the $< n$ genes considered for addition by the greedy algorithm) the gene g added to S by the greedy algorithm in iteration i is not in $H \setminus S$ with probability $\leq e^{-\frac{2m^2}{4mk^2(c''')^2}}$ which is $\leq \delta/k$ when $m \in \Omega\left(k^2 \ln(nk/\delta)\right)$. □
